# An equivalent circuit model for onset and offset exercise response

**DOI:** 10.1186/1475-925X-13-145

**Published:** 2014-10-18

**Authors:** Yi Zhang, Azzam Haddad, Steven W Su, Branko G Celler, Aaron J Coutts, Rob Duffield, Cheyne E Donges, Hung T Nguyen

**Affiliations:** The Faculty of Aeronautics and Astronautics, University of Electronic Science and Technology of China, 611731 Chengdu, China; The Faculty of Engineering and IT, University of Technology, 2007 Sydney, Australia; CSIRO ICT Centre, Sydney, Australia; School of Human Movement Studies, Charles Sturt University, Bathurst, Australia

**Keywords:** Heart rate, Oxygen uptake, Mathematical modeling, Cardiovascular system, Single-cycle square wave, Interval training

## Abstract

**Background:**

The switching exercise (e.g., Interval Training) has been a commonly used exercise protocol nowadays for the enhancement of exerciser’s cardiovascular fitness. The current difficulty for simulating human onset and offset exercise responses regarding the switching exercise is to ensure the continuity of the outputs during onset-offset switching, as well as to accommodate the exercise intensities at both onset and offset of exercise.

**Methods:**

Twenty-one untrained healthy subjects performed treadmill trials following both single switching exercise (e.g., single-cycle square wave protocol) and repetitive switching exercise (e.g., interval training protocol). During exercise, heart rate (HR) and oxygen uptake (VO _2_) were monitored and recorded by a portable gas analyzer (K4b ^2^, Cosmed). An equivalent single-supply switching resistance-capacitor (RC) circuit model was proposed to accommodate the observed variations of the onset and offset dynamics. The single-cycle square wave protocol was utilized to investigate the respective dynamics at onset and offset of exercise with the aerobic zone of approximate 70% - 77% of HR _*max*_, and verify the adaption feature for the accommodation of different exercise strengths. The design of the interval training protocol was to verify the transient properties during onset-offset switching. A verification method including Root-mean-square-error (RMSE) and correlation coefficient, was introduced for comparisons between the measured data and model outputs.

**Results:**

The experimental results from single-cycle square wave exercises clearly confirm that the onset and offset characteristics for both HR and VO _2_ are distinctly different. Based on the experimental data for both single and repetitive square wave exercise protocols, the proposed model was then presented to simulate the onset and offset exercise responses, which were well correlated indicating good agreement with observations.

**Conclusions:**

Compared with existing works, this model can accommodate the different exercise strengths at both onset and offset of exercise, while also depicting human onset and offset exercise responses, and guarantee the continuity of outputs during onset-offset switching. A unique adaption feature by allowing the time constant

(Continued on next page) (Continued from previous page)

and steady state gain to re-shift back to their original states, more closely mimics the different exercise strengths during normal daily exercise activities.

## Background

One of the greatest public health challenges confronting many industrialised countries is the obesity epidemic. Low-to-moderate intensity exercise, suitable for every fitness level, remains one of the healthiest and risk averse methods for reducing body fat [1]. Heart rate (HR) and oxygen uptake (VO _2_) are commonly applied to assess metabolic demands [2–7]. To develop an effective exercise protocol to improve human cardiovascular fitness, this study first explores the dynamic responses of HR and VO _2_ by using a portable gas analyzer (K4b ^2^, Cosmed) during treadmill experiments. Twenty-one untrained healthy subjects performed treadmill exercise following the predefined single-cycle square wave and interval training protocols. The single-cycle square wave protocol was utilized to investigate the respective dynamics at onset and offset of exercise with a certain submaximal exercise capacity (an approximate range of 70% - 77% of HR _*max*_, or 56% - 65% of VO _2*max*_ [8]). Additionally, an interval training protocol [9] is generally inclusive of three different periods: warm-up, exercise (three-cycle of high intensity period and recovery period), and cool-down. The design of the interval training protocol regarding this study was to verify the transient properties during onset-offset switching.

Previous literatures [10–12] have studied human cardiorespiratory responses at onset and offset of exercise, and found the different dynamic characteristics (i.e., time constants and steady state gains) at onset and offset of exercise. We further explored dynamics in the particular aerobic zone (approximate 70% - 77% of HR _*max*_, or 56% - 65% of VO _2*max*_ [8]), which has well confirmed the observation reported in literatures [13]. Past works also focused on building a model for estimates of HR and/or VO _2_ responses to exercise. See [14–21] for examples. These models utilized only a single non-switching model for either onset or offset exercises. The traces of onset and/or offset dynamics would have been accurately described but the transient properties during onset-offset switching are almost overlooked. Switching models produce much better results than single non-switching models. The switching resistance-capacitor (RC) circuit introduced by [13] used a dual-supply threshold-based solution to simulate HR and VO _2_ responses towards the interval training protocol. Despite a better performance being observed (vs. the non-switching models), particularly for transient behaviors during switching, there are still some limitations since dynamical characteristics (i.e., time constant and steady state gain) of model are not allowed to re-shift back to their original states, especially at the offset of exercise.

In this paper we propose an innovative single-supply switching RC circuit model. This will depict and analyze HR and VO _2_ dynamics to exercise, consisting of only one power supply, linked with onset and offset RC switching circuits. The main advantages of this model are that it can well accommodate the observed onset and offset dynamics, guarantee the continuity of model outputs during switching, and adaptively match the measured output for different exercise strengths at both onset and offset of exercise.

The list of nomenclature information is included in Table 1. The remainder of the paper is organized as follows. Section ‘Experiment’ introduces experimental equipment, exercise procedures and protocols. Section ‘Data analysis’ shows the data analysis for parameter identification of the proposed model. Section ‘The proposed modeling and verification methods’ describes the proposed single-supply switching RC circuit model and its verification methods. Section ‘Results’ provides the parameter configuration, verifications, and discussions. Finally, Section ‘Conclusion’ concludes this study.

**Table 1:** **The list of abbreviations and terms**

Abbreviation	Nomenclature
HR	Heart rate
HR _*max*_	Maximum heart rate
VO _2_	Oxygen consumption
VO _2*max*_	Maximum oxygen consumption
RC	Resistance-capacitor
RMSE	Root-mean-square-error
UTS	University of Technology, Sydney
DPDT	Double-pole double-throw
SV	Stroke volume
ATP	Adenosine triphosphate
bpm	Beat per minute
STD	Standard deviation
Q	Cardiac output
V	Power supply
DC	Direct current

## Experiment

In order to investigate HR and VO _2_ responses with a certain submaximal exercise capacity [8], twenty-one male healthy untrained subjects participated in the single-cycle square wave and interval training exercises. The UTS Human Research Ethics Committee (UTS HREC 2009000227) approved this study and an informed consent was obtained from all participants before commencement of data collection. The physical characteristics of the participants joined the single-cycle square wave exercise are presented in Table 2.

**Table 2:** **Subject physical characteristics**

Subject	Age (year)	Height (cm)	Mass (kg)
ANDW	27	175	55
AHMD	32	170	87
ISSA	29	176	90
YASA	29	187	100
ARDI	42	175	80
RAMI	29	164	64
SATM	31	169	67
OMAR	26	180	77
ANEL	40	173	102
BIKE	45	179	97
BRRU	45	173	101
CHRI	37	170	71
DACR	53	183	99
GAHI	45	182	98
MABR	36	186	92
MACU	53	175	89
MAYE	45	180	94
RABL	43	178	100
ROMU	50	182	86
WADO	53	173	73
**MEAN**	38.02	175.69	83.27
**STD**	5.28	4.98	10.80

STD: Standard Deviation.

Prior nutritional intake, physical activity and environment conditions were standardized for all participants. The participants consumed a standardized light meal at least two hours before the experiment and were not to engage in any exercises for one day prior to each experiment [22, 23]. The temperature and humidity of the laboratory were set at 20 - 25°C and 50% relative humidity, respectively.

**Figure 1:**
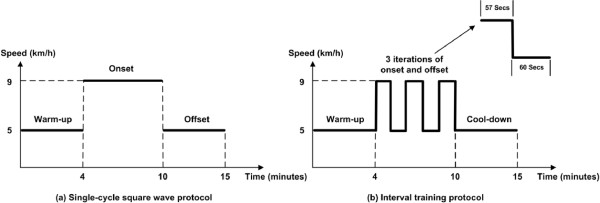
**The predefined exercise protocols in connection to exercise intensities and time durations of warm-up, onset and offset segments.** The left axis **(a)** indicates the proposed single-cycle square wave protocol following warm-up at 5 km/h for 4 minutes, onset running at 9 km/h for 6 minutes and offset walking at 5 km/h for at least 5 minutes. The right axis **(b)** indicates the proposed interval training protocol following warm-up at 5 km/h for 4 minutes, 3 iterations of onset at 9 km/h for 57 seconds and offset at 5 km/h for 60 seconds, and cool-down at 5 km/h for at least 5 minutes.

**Figure 2:**
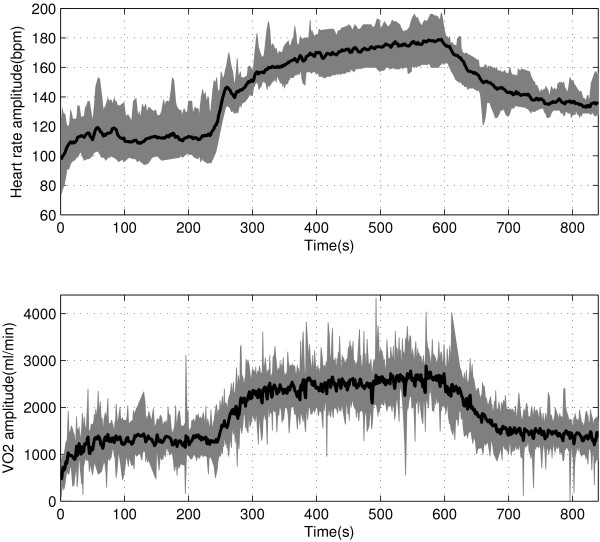
**The HR and VO**_**2**_ **experimental data for twenty subjects following the predefined single-cycle square wave protocol.** Thick black lines indicate the average response of HR and VO _2_ and the gray surface indicates plus and minus deviations over forty trials of twenty subjects.

The step responses of HR and VO _2_ at onset and offset of exercise were measured following the predefined two protocols: the single-cycle square wave and interval training protocols. Figure 1 shows the exercise intensities and durations of these exercise protocols. The single-cycle square wave protocol (see Figure 1a) was repetitively performed by twenty subjects for minimizing effects of the intra subject variability. The inter subject variability (e.g., the fast response of vagal withdrawal, sudden increase of body temperature, nervousness at the start of exercise) was as well considered through the initiating warm-up, asking subjects to gently walk on the treadmill with 5 km/h before the onset of the experiment. Figure 2 shows a typical experiment result of the ensemble averages of HR and VO _2_ responses following such protocol across twenty subjects. To explore the transient behaviors during onset and offset of exercise, a new male subject AZAM (Age = 30 year, Height = 185 cm, and Mass = 84 kg) was invited to run on the treadmill following the interval training protocol, proposed in Figure 1b.

In order to investigate cardiorespiratory responses to the moderate exercise intensity level, the aerobic zone of approximate 70% - 77% of HR _*max*_ (or 56% - 65% of VO _2*max*_) was targeted for exercisers following both exercise protocols [8], since the relationship between HR and VO _2_ in this zone is nearly linear [24]. To determine HR _*max*_ for any individual subject, the equation employed for this study was developed by Inbar [25]:
1

All physiological measurements in this study were collected by a Cosmed portable gas analyzer (K4b ^2^, Cosmed, Rome, Italy). The Cosmed system includes a compatible HR monitor which consists of one transmitter in the elastic belt and one receiver. The two parts are assembled as close as possible for capturing the most effective communication signals. K4b ^2^ gas analyzer and its compatible products are chosen because they have been reported to be valid, accurate and reliable [26–28]. To avoid random errors and improve the accuracy of the recorded data, each exercise was repeated twice by subjects and the obtained data filtered, interpolated, and averaged.

## Data analysis

It has been widely known that the step responses of HR and VO _2_ can be approximated as a first-order process [29], , where *K* is the steady state gain and *T* is the time constant. On the basis of the experimental data of the single-cycle square wave protocol Matlab System Identification Toolbox was used to establish the first-order process for both HR and VO _2_ responses over all trials. The coefficients (*K* and *T*) for each trial are identified, and the mean and standard deviation (STD) of twenty subjects at onset and offset of exercise are illustrated in Table 3. Those results indicate that the steady state gain (*K*) at offset of exercise is obviously smaller than that at onset of exercise for both HR and VO _2_. The mean values of time constant (*T*) at offset of exercise, however, is notably larger than that at onset for both HR and VO _2_.

**Table 3:** **The mean and STD results of** ***T*** **and** ***K*** **of the experiment results for the HR and VO**_**2**_ **responses at onset and offset of exercise**

Coefficients	HR		VO _2_	
	Mean	STD	Mean	STD
*T*_*on*_(sec)	60.60	17.52	54.97	10.78
*K*_*on*_	13.35	2.26	356.43	60.12
*T*_*off*_(sec)	88.99	39.08	65.90	13.43
*K*_*off*_	10.15	1.87	340.30	57.93

*T*_*on*_, *T*_*off*_, *K*_*on*_ and *K*_*off*_ represent time constant (T) and steady state gain (K) at onset and offset of exercise respectively.

## The proposed modeling and verification methods

### The single-supply switching RC circuit model

**Figure 3:**
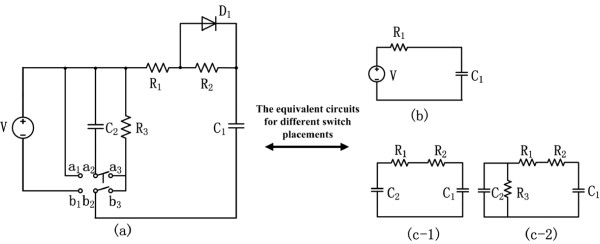
**The single-supply switching RC circuit model for cardiorespiratory responses at onset and offset of exercise. (a)**. the proposed circuit model for both onset and offset of exercise; **(b)**. the onset subcircuit; **(c-1)**. the offset subcircuit c-1; **(c-2)**. the offset subcircuit c-2.

Figure 3a shows the overview of the proposed single-supply switching RC circuit model, which is inclusive of one DC power supply (*V*), one diode, one double-pole double-throw (DPDT) switch, two capacitors (*C*_1_ and *C*_2_), and three resistors (*R*_1_, *R*_2_ and *R*_3_). Figures 3b and c-1/c-2 are the subcircuits of the proposed model linked by the DPDT switch representing cardiorespiratory behaviors at onset and offset, respectively. The voltage of *C*_1_ with respect to exercise time represents the amplitude of HR and/or VO _2_ dynamics during moderate exercise and its subsequent recoveries, since in moderate exercise both HR and VO _2_ have similar behaviors [13, 24]. The functionality of D _1_ is to configure the resistance amplitude of the onset and offset circuits, which will short R _2_ off during the activation of the onset circuit. The process of modeling both HR and VO _2_ dynamics at onset and offset of exercise and long-term recovery is as follows.

**Figure 4:**
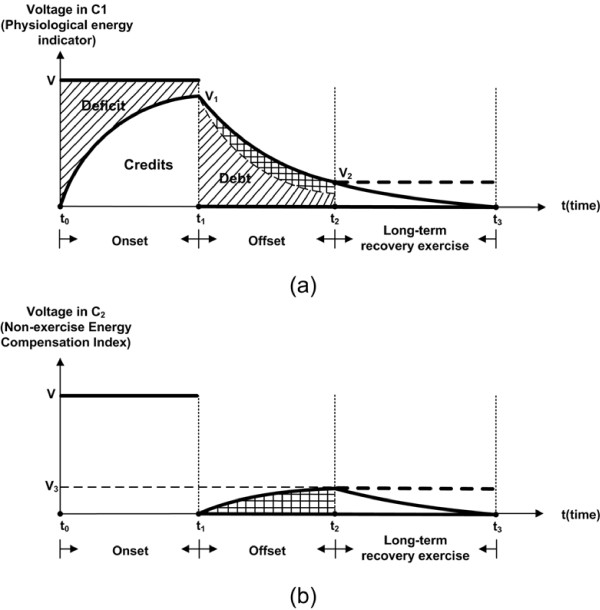
**Voltage variations of capacitors** ***C***_**1**_ **and** ***C***_**2**_ **of the proposed model at onset, offset and long-term recovery exercise. (a)**. the voltage variation of C _1_; **(b)**. the voltage variation of C _2_.

At first, the onset behaviors are simulated by switching DPDT to poles *a*_1_ and *b*_1_, (see Figure 3b). The function of the dioxide *D*_1_ is to short the *R*_2_ out. In this period, the DC power supply *V* charges the capacitor *C*_1_, from baseline up to *V*_1_ that approximately equals the DC power supply *V*. Figure 4 shows the dynamic variations of capacitors *C*_1_ and *C*_2_ in the proposed model during exercise and recovery. The voltage of *C*_1_ is expressed as:
2

where the steady state value of V is known as *V*.

During the offset period from *t*_1_ to *t*_2_ (see Figure 4), both circuits c-1 and c-2 would be applicable for the analysis of this period. However, if assume *R*_3_ is sufficiently big, the current passing through *R*_3_ would be negligible, meaning that both circuits (c-1 and c-2) with such assumption for *R*_3_ are approximately equivalent. The offset processes for *C*_1_ and *C*_2_ can be described as:
34

during which the capacitor *C*_1_ is discharging and its voltage follows an exponential decay down to *V*_2_ at time *t*_2_, while the capacitor *C*_2_ is charging resulting in an exponential growth of its voltage from 0 at time *t*_1_ to *V*_3_ at time *t*_2_. It is also required that  at the end time of offset portion, *t*_2_.

The particular offset dynamics of *C*_1_ was intended to mimic a repetitive switching training behavior (e.g., interval training [30]). At this stage, the steady state level of *C*_1_ would shift from a high level (e.g., *V*_1_) to a low level (e.g., *V*_2_) comparing to the initiating level at warm-up (called the baseline level) herein being considered as zero. The high- and low- levels can easily implement by manipulating the amplitudes of resistances and capacitors of the proposed model. Considering the single switching exercise (e.g., a single-cycle square wave exercise introduced in Section ‘Experiment’), however, the steady state level must re-shift back to the baseline since the human metabolic rates will generally return back to their baseline levels during the long-term recovery. It could be well achieved by setting the model with the alternative subcircuit c-2, which can consume all energies stored in capacitors *C*_1_ and *C*_2_ through the resistance *R*_3_. Figure 4 shows this long-term recovery process where the *C*_1_ and *C*_2_ voltages fall down to the baseline at time *t*_3_.

Based on equations (2)-(4), the normalized time constants and steady state gains for both onset and offset processes could be derived as follows:
5

where *K*_*on*_, *T*_*on*_, *K*_*off*_, and *T*_*off*_ represent the steady state gains and the time constants of onset and offset respectively. New defined parameters  and  are applied to normalize steady state gains.

If *K*_*on*_, *K*_*off*_, *T*_*on*_, and *T*_*off*_ are given and assume *R*_2_ is a pre-defined free parameter, the values of capacitors and resistor (*C*_1_, *C*_2_, and *R*_1_) then could be easily configured by:
6

### Quantitative description for the concept of ‘oxygen debt’

The physiological interpretation for the dynamics of HR/VO _2_ responses at onset and offset of exercise may be associated with the term ‘oxygen debt’, as first coined by A. V. Hill and others [31]. According to the term ‘oxygen debt’ [31], the body’s carbohydrate stores are linked to energy ‘credits’. If these stored credits are expended during onset of exercise, then a ‘debt’ is occurred. The greater energy ‘deficit’, or use of available stored energy credits, the larger energy ‘debt’ occurs [10]. The ongoing oxygen uptake after onset of exercise is then thought to represent the metabolic cost of repaying this debt. This concept used financial-accounting terms to qualify exercise metabolism; in fact, it is still popularized to the day.

Moreover, this study attempts to develop an electronic term to quantitatively analyze the switching exercise processes. First of all, the onset circuit could well support the hypothesis made by the term ‘oxygen debt’ [31]. During this period shown in Figure 4, *V*_*c*1_(*t*) exponentially grows implying an increase of HR. It has been well known that the cardiac output (Q), the total power pumped by the heart, can be expressed as Q = stroke volume (SV) × HR. As during moderate exercises SV is assumed to be constant, the integral of HR with respect to time should be proportional to Q, which also can be depicted by the integral of equation 2, see the white area of the onset period in Figure 4(a). In the concept of ‘oxygen debt’, this white area is thought as energy ‘credits’, and the line shadowed area is considered as energy ‘deficit’ representing the amount of ATPs that are not capable to be pumped out to satisfy the tissue’s urgent demands. Similar with the proposed circuit model, a simply RC serial circuit is employed for approximations of the onset dynamics. Since *V*_*c*1_(*t*) cannot instantaneously reach to the steady state level (*V*) at the beginning of exercise, energy ’credits’ and ‘deficit’ occur.

Currently, the precise biochemical explanation for offset of exercise is not possible because the specific chemical dynamics are still unclear [10]. A. V. Hill [31] first hypothesized that all energies generated during the offset period (the line shadowed area plus the cross line shadowed area between *t*_1_ and *t*_2_ in Figure 4(a)) are thought to represent the metabolic cost of repaying energy ‘debt’. However, this study proves that the amount of energy ‘debt’ is much larger than that of energy ‘deficit’, which means energy ‘debt’ is only a part of energies generated during the offset period. Instead, glycogenesis and all other processes related for the recovery of the body to its pre-exercise conditions also are taking place in the offset period.

The experimental observation (see section ‘Experiment’) has shown that the time constant at offset of exercise is larger than that at onset of exercise, meaning that the line shadowed area plus the cross line shadowed area in the offset period (see Figure 4(a)) is greater than the area of energy ‘deficit’ in the onset period. If the two line shadowed areas (the areas of energy ‘deficit’ and ‘debt’ in Figure 4(a)) could equal each other (the debt equals to the deficit), a question is raised: what does the extra area (the cross line shadowed area in Figure 4(a)) represents? According to the mass-energy equivalence relation (*E*=*MC*^2^), any change in the energy of an object causes a change in the mass of that object. Thus, the extra cross line shadowed area perhaps implies there must exist an energy storage process, which converts the energy into ‘molecules’, and further causes a change in the body’s mass. As the specific chemical dynamics are still unclear [10], it might be safely concluded that any physiological process that contributes to the recovery of the body to its pre-exercise conditions may result in the appearance of such extra area, e.g., glycogenesis (a process of glycogen synthesis). For this reason, it is probably that the proposed element *C*_2_ is going to store this kind of energy, like the liver stores glycogen. Overall, the model outputs indicate that the cross line shadowed area in Figure 4(b) is presumably equal to the one with the same mark in Figure 4(a).

### Model verification

In order to verify the proposed modeling work, two independent and widely used metrics were used for comparative purposes. Root-mean-square-error (RMSE), as described in Equation (7), was calculated to provide a measure of the average error between the two waveforms.
7

where *x*_1,*i*_ and *x*_2,*i*_ are the *i*th sample from measured data and model output respectively and *n* is the number of samples. Correlation coefficient, as described in Equation (8), was used to provide a measure of the similarity in the shape of the model outputs versus the averaged experiment results.
8

where *P*_1_ and *P*_2_ are the measured and estimated data in terms of HR and VO _2_ response at onset and offset of exercise respectively.

## Results

### Parameter configuration

Based on the dynamic characteristics of observed HR and VO _2_ and normalization process shown in Table 3 and Equations (5) - (6) respectively, the tuned circuit model parameters for the proposed single-supply switching RC circuit model were demonstrated in Table 4. The tuned averaged models for both HR and VO _2_ then were employed to simulate those dynamic variations following both single-cycle square wave and interval training protocols. The simulation was performed by the Matlab/Simulink module, and the timing of DPDT switching between exercise intensities strictly follows the reference protocols shown in Figure 1.

**Table 4:** **Parameter determination of the proposed single-supply switching RC circuit model for both averaged HR and VO**_**2**_ **dynamics at onset and offset of exercise for twenty subjects**

Model parameters	HR	VO _***2***_
V (*V*)	55.1	1319
R _1_ (*Ω*)	100.9028	248.3758
R _2_ (*Ω*)	100	100
C _1_ (*F*)	0.6430	0.1916
C _2_ (*F*)	1.7782	3.8900

### Comparison for single-cycle square wave protocol

**Figure 5:**
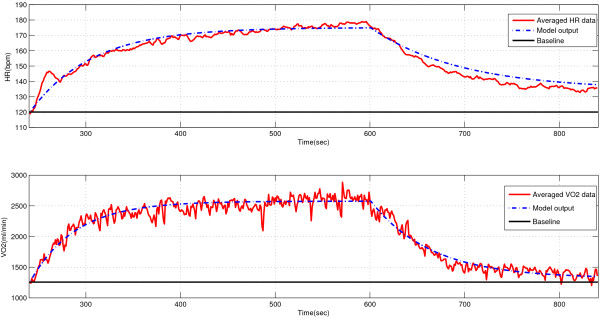
**The model outputs vs. the averaged experimental data of HR and VO**_**2**_ **dynamics at onset and offset of exercise.** The bold lines indicate the baseline of steady state level and closely reflect metabolic rates at warm-up. The red curves indicate HR and VO _2_ averaged data from experimental observations over twenty subjects. The dashed blue curves indicate the model outputs based on the determined parameters.

Figure 5 was an example to show the model accuracy towards the single-cycle square wave protocol. The proposed model was first tuned by settings of parameters based on Table 4, then run in Matlab/simulink following proposed duty cycles of the predefined single-cycle square wave protocol. The mean and variance of the distributions can be found in Table 5. The RMSE of HR and VO _2_ across the general average measurements of all subjects for the proposed model was 3.13 *bpm* and 97.35 *ml*/*min* respectively. The correlation coefficient between the actual measurements and model estimations was 98.11% and 97.98%. It can be seen in Figure 5 that the proposed model significantly performed the estimation of HR and VO _2_ dynamics for an averaged general-population set following single switching exercise protocols.

**Table 5:** **Statistics of correlations between actual data and model outputs**

Protocols	HR		VO _2_	
	***μ***(%)	***σ***	***μ***(%)	***σ***
Single switching exercise	98.11	3.13	97.98	97.35
Repetitive switching exercise	97.34	2.84	83.85	234.42

*μ* and *σ* are the correlation coefficient and RMSE respectively.

### Comparison for interval training protocol

**Figure 6:**
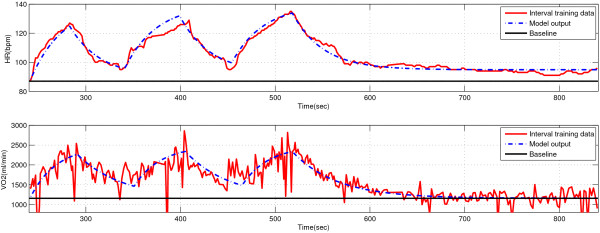
**The model outputs vs. the experimental data of HR and VO**_**2**_ **dynamics for subject AZAM under the predefined interval training protocol.**

The subject AZAM was invited to perform the predefined interval training regarding the model verification for the repetitive switching exercise. Experimental results for both HR and VO _2_ dynamics are shown in red curves of Figure 6. Of the three-cycle interval training exercise, model parameters were determined by using the first cycle measurement and those tuned circuit models for onset and offset exercises were accurately switched following the predefined protocol durations illustrated in Figure 1b. The dashed blue curves in Figure 6 indicate the model outputs of proposed interval training exercise for both HR and VO _2_ dynamics of subject AZAM.

When comparing the model accuracy versus the observations from the specific-subject data following the repetitive switching exercise, based on correlation coefficients shown in Table 5, the model outputs can generally describe the dynamics of HR and VO _2_ with a high similarity (97.34% and 83.85%, respectively). When the RMSE for HR and VO _2_ was examined, it was evident that the model output for HR again were fairly accurate but that for VO _2_ had errors with 234.42. This was primarily due to the presence of random errors, which caused more variability of the repetitive exercise in the specific-subject data versus the averaged general-population data.

### Discussion

This model was tested through those exercise protocols with few iterations of onset and offset periods, but even with more iterations, it enables estimates of the dynamic response of HR and VO _2_. The employed switching mechanism could well unify the difference at onset and offset of exercise, as well as satisfy the requirement of the continuity of model outputs during switching. This feature results in an accurately quantitative analysis for human exercise responses, and can further apply to regulating and improving cardiorespiratory fitness.

**Figure 7:**
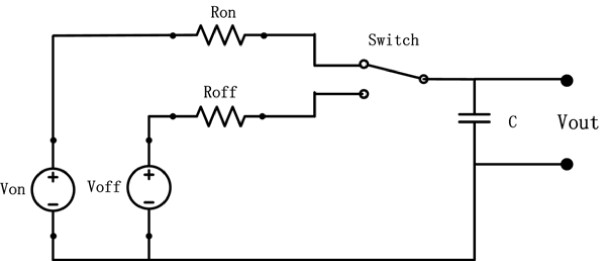
**The RC circuit introduced by Azzam et al., where the voltage of capacitor** ***C*** **was used to simulate the HR and VO**_**2**_ **dynamics towards interval training protocols,** ***V***_***on***_ **,** ***R***_***on***_ **,** ***V***_***off***_ **, and** ***R***_***off***_ **are the onset and offset supplies and resistances respectively that were switched by a designed SPDT switch.**

Currently, Azzam et al. developed a dual-supply threshold-based solution to simulate HR and VO _2_ responses towards the interval training protocols, which employs dual power supplies to set a threshold value for each onset and offset scenario [13]. Figure 7 shows the RC circuit introduced by Azzam et al. Although this model can well describe the switching properties during onset and offset of exercise, there are still some limitations since dynamical characteristics (i.e., time constant and steady state gain) of model are not allowed to re-shift back to their original states due to the effect of *V*_*off*_. It is probably inefficient when applying it to a single switching exercise, as it requires the metabolic rate can adaptively vary from *V*_1_ down to zero (see Figure 4). Compared with the one shown in Figure 7, the proposed model provided sound results for both single and repetitive switching exercises.

Further investigation would be made to explore the subject-specific model across a population of individuals, although it has been found the proposed model can work on the averaged experimental observations with acceptable correlations.

Moreover, to regulate the proposed switching model the implementation of bump-less switching between two or more higher dimensional systems based on multi-realization theory will also be discussed in the next step [32, 33].

## Conclusion

In this work a novel single-supply switching RC circuit model is presented to accommodate the variations of onset and offset dynamics following both single-cycle square wave and interval training protocols. Twenty-one healthy untrained subjects were invited to participant the treadmill exercises. The portable gas analyzer K4b ^2^ was used to measure breath-by-breath VO _2_ and beat-by-beat HR values. It has been concluded that the observed results can be reliably described by the proposed model. Unlike some other existing modeling works, it provided accurate analyses for the different responses of onset and offset exercises, guaranteed the continuity of model outputs during onset-offset switching, and is capable of accommodating exercise strengths. The validity of the proposed model is confirmed by comparing the simulated model outputs with the averaged experimental observations. In the next step, a subject-specific model will be investigated and a general framework for the implementation of bump-less switching between two or more higher dimensional systems based on multi-realization theory [32, 33] then will be developed for the issue of human exercise regulation.
